# Parenting Styles, Food Parenting Practices and Dietary Intakes of Preschoolers

**DOI:** 10.3390/nu13103630

**Published:** 2021-10-16

**Authors:** Biyi Chen, Kendra Kattelmann, Christopher Comstock, Lacey McCormack, Howard Wey, Jessica Meendering

**Affiliations:** 1 School of Health and Consumer Sciences, South Dakota State University, Brookings, SD 57006, USA; biyi.chen@jacks.sdstate.edu (B.C.); christopher.comstock@sdstate.edu (C.C.); lacey.mccormack@sdstate.edu (L.M.); jessica.meendering@sdstate.edu (J.M.); 2 Ethel Austin Martin Program, South Dakota State University, Brookings, SD 57006, USA; howard.wey@sdstate.edu

**Keywords:** parenting styles, food parenting practices, dietary intakes, preschoolers

## Abstract

Previous evidence suggests that children’s eating behaviors were largely influenced by the parent and home eating structure. This study examined the relationship between parenting styles (including authoritative, authoritarian, indulgent, and uninvolved), food parenting practices (within Structure, Coercive Control, and Autonomy Support constructs) and dietary intakes of preschoolers. Children aged 3–5 years and their parents were recruited from preschools/daycare centers and parents completed the surveys (*n* = 166). Dietary intakes were collected using the Harvard Service Food Frequency Questionnaire (HSFFQ), parenting style was assessed using the Parenting Dimensions Inventory-Short Version (PDI-S), and food parenting practices were measured using Comprehensive Home Environment Survey (CHES). The results showed that food parenting practices had a higher number of specific significant findings on children’s nutrient and food group intakes than parenting styles. Correlation analyses showed positive parenting practices within Structure were significantly related to healthier children’s intakes (e.g., vegetables, iron, and folate) and less unhealthy dietary intakes (e.g., sweets and total fats). Regression models show that children with authoritative parents consumed more fruits compared to children with authoritarian parents and indulgent parents. The results addressed the importance of parental influences for preschoolers’ healthy dietary intakes, which suggested that future interventions and educational programs could enhance parenting practices to impact child diet.

## 1. Introduction

A growing body of evidence suggests that children’s eating behaviors are largely influenced by their parents and their home eating structure [1,2], which set the stage for future habits and may influence a child’s weight status [3]. The link between the parent and the weight status of a child becomes especially important when one considers that children who are overweight and obese are more likely to become adults with obesity [4,5], and children with obesity are at an increased risk of chronic disease later on in life [6,7,8]. Establishing healthy eating and exercise habits at an early age is crucial in preventing unnecessary weight gain.

Preschool age is a critical development period for adopting healthy, behaviors because eating habits and preferences established during this time are likely to continue through childhood and into adulthood [9]. The overall diet quality among U.S. preschool children is low [10], as most do not meet the recommended intakes for vegetables and whole grains, and exceed the recommended intakes of refined grains, solid fats, and added sugars [11]. Parental influences play a particularly important role in shaping dietary behaviors in young children [12]. For preschoolers, parents are usually the primary determinants of the types and the amount of food that children eat [13].

Children’s dietary intakes could be influenced by their parents’ general parenting approach (i.e., parenting styles) or by specific parenting practices (e.g., food parenting practices) [14]. Parenting style refers to the parenting across situations and reflects on the emotional climate in which children are raised [14]. In previous studies, some have shown that parenting styles influence children’s dietary behaviors and weight status [9,15,16,17,18], but others have not found any relationship between these [19,20]. Authoritative and authoritarian styles have been widely investigated in relation to childhood obesity [15]. Two previous systematic reviews concluded that children raised in authoritative households ate more healthily, and authoritative parenting was associated with a healthier BMI [16,17]. Other studies also reported that an authoritative style was positively associated with parental attempts to get the child to eat dairy, fruit, and vegetables [21,22].

Parenting practices are “behaviors defined by specific content and socialization goals” [23]. A recent study found that a number of parenting practices were linked to improved child diet quality [1], including encouragement/modeling of healthy eating, making healthful foods readily available in the home, and setting home food rules on snacking type, place, and size [1]. While there is a wealth of evidence on general parenting practices, child nutrition researchers have recently begun studying food-related parenting practices and their relationships with child diet and eating behaviors [24]. These practices are referred to as food parenting practices. Vaughn’s parenting practices content map [24] details three food parenting practice constructs—Structure, Coercive Control, and Autonomy Support—and specific subconstructs within each construct. In the literature, food parenting practices related to Structure (e.g., rules and limit, meal and snack routine, and food availability and accessibility) and Autonomy Support (e.g., encouragement and nutrition education) were associated with a higher intake of fruits and vegetables and a lower intake of unhealthy foods (e.g., sugar-sweetened beverages, snacks, and sweets) [24,25]. Coercive Control practices (e.g., restriction, pressure to eat, and threats and bribes) were found to have inverse associations [24,25].

Even though the relationships between parenting styles/parenting practices and child eating have been explored, limited research includes both parenting styles and food parenting practices when examining how parents may influence preschoolers’ diets [26]. Including parenting styles or food parenting practices separately does not adequately describe how children experience mealtimes with their parents [27]. Some studies have only focused on the general parenting style, but this may be inappropriate, because parenting style is more specific to the eating environment [28]. A previous study showed that the role of general parenting style in the prediction of children’s dietary intake was minimal, which might be because of the general parenting style as operationalized in the study is too broadly defined and therefore insensitive to differences in dietary habits [29]. Thus, this study is necessary to consider the influences of both parenting styles and food parenting practices on children’s dietary intakes, which mimics what children would experience at mealtimes.

**Figure 1:**
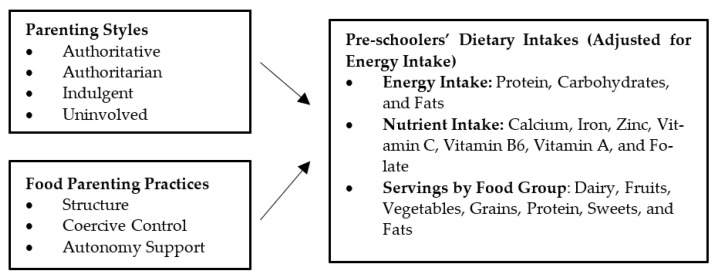
The current study model of parenting styles, food parenting practices and preschoolers’ dietary intakes.

Therefore, the purpose of this study was to examine the relationship between parenting styles/food parenting practices and dietary intakes of preschool-aged children. Figure 1 illustrates the model assessed in this study. Specifically, it was hypothesized that the authoritative parenting style and the positive food parenting practices (e.g., subconstructs within Structure and Autonomy Support), would be related to healthier children’s dietary intakes (e.g., protein, selected nutrients, fruits, and vegetables) or less unhealthy dietary intakes (e.g., sweets and fats) than the other three parenting styles and negative food parenting practices (e.g., subconstructs within Coercive Control).

## 2. Materials and Methods

This study was a secondary data analysis of the *iGrow* program conducted in 2016, which was an educational literacy curriculum aiming to teach preschool children the benefits of making healthy decisions involving nutrition and physical activity. Institutional Review Board (IRB) approval, child assent, and parental consent were obtained in accordance with the policy statements of the Human Subjects Committee at South Dakota State University.

### 2.1. Participants

Children aged 3–5 years and their parents were recruited from preschools/daycare centers in South Dakota, Minnesota, and Nebraska. To qualify for recruitment, preschools/daycare centers needed to meet the following selection criteria: (1) have at least two classrooms with 3–5 year old children, and (2) have separate teachers in each classroom. In total, 14 preschools and daycare centers met the selection criteria and agreed to participate in the study. From the 14 participating preschools and daycare centers, 293 parent/child dyads were recruited via email and with flyers describing individual eligibility and the benefits of participation. The final sample used in this study consisted of 166 dyads. 

### 2.2. Data Measurements

#### 2.2.1. Demographic Information

Demographic information regarding age, race, household income, and education was self-reported by parents. Height and weight of children and parents were measured in duplicate by trained research assistants to the nearest 0.1 cm and 0.1 kg, respectively. Parent Body Mass Index (BMI) categories and Child BMI-for-age percentiles were calculated according to Centers for Disease Control and Prevention guidelines [30,31].

For analytic purposes, there were the combinations of response scale to fewer groups for demographic variables, which are described as follows: 4 categories of BMI were dichotomized as normal/underweight and overweight/obese; 12 categories of household income were dichotomized as <$60,000 and $60,000 or greater; 6 categories of parent education were dichotomized as associate’s degree or less and bachelor’s degree or higher; and due to the low response numbers for non-white groups, 6 categories of race were dichotomized as white and non-white.

#### 2.2.2. Measures of Dietary Intakes

Dietary information was collected using the Harvard Service Food Frequency Questionnaire (HSFFQ). The questionnaire lists 86 different food items and asks for the number of times the food was consumed over the past 4 weeks [32,33]. The HSFFQ has been validated for the assessment of nutrient intake among children of 1 to 5 years of age [34]. The questionnaire was completed by caregivers on behalf of their children. The HSFFQ User’s Manual [35] was used for guiding the analysis. In general, portion size in gram weight assigned to each food item on the HSFFQ was derived from national data [35,36]. Nutrient data per 100 gm portion for the food items were derived from the HSFFQ and were estimated using the Harvard nutrient database [36]. Daily nutrient intakes from the HSFFQ were computed by converting the food frequency to a daily number of servings to each food item, multiplying this by the nutrient content for the assigned weight, and summing the value for all foods [35]. Food groups (dairy, fruits, vegetables, grains, protein, sweets, and fats) were determined by the HSFFQ [35]. (Complete instructions were described in Supplementary Materials File 1).

The HSFFQ provided dietary intakes results regarding daily energy and macronutrient intake (carbohydrates, protein, and fats), selected micronutrient intake (vitamin A, vitamin C, calcium, zinc, folate, iron, protein, and vitamin B6), and the number of servings from food groups (protein, grain, dairy, vegetables, fruit, sweets, and fats). Energy, macronutrient, and micronutrient intake, were calculated from the frequency reported and the nutrients in the corresponding servings of food. The food group intake was a direct reporting of the number of servings that the participants reported.

#### 2.2.3. Measures of Parenting Style

Parenting style was assessed using the Parenting Dimensions Inventory-Short Version (PDI-S) and was completed by parents [37]. The short version of PDI retains the most reliable and valid components and can be used for preschoolers [37]. In this study, two dimensions of the PDI-S, including nurturance (6 items, Cronbach’s alpha = 0.80) and amount of control (5 items, Cronbach’s alpha = 0.71), were used to classify parents into four parenting styles [37,38] (Supplementary Materials Table S1). Parents were categorized into parenting style groups by conducting a cross-classification of high and low median scores on the dimensions of nurturance and the amount of control. Four parenting styles include authoritative (high on both nurturance and amount of control), authoritarian (low on nurturance and high on amount of control), indulgent (high on nurturance and low on amount of control), and uninvolved (low on both nurturance and amount of control) [38,39].

#### 2.2.4. Measures of Food Parenting Practices

Food parenting practices were measured using a Comprehensive Home Environment Survey (CHES), which is a reliable and validated comprehensive measurement related to the home environment and parenting behaviors [40]. Large numbers of items in this comprehensive tool were related to food parenting practices; however, they were not identified. Thus, the research team identified and added new factor structures to the CHES by conducting an exploratory factor analysis. Full details of the analysis have been previously described [41]. Three broad food parenting practice constructs were identified, including Structure (Cronbach’s alpha = 0.78), Coercive Control (Cronbach’s alpha = 0.73), and Autonomy Support (Cronbach’s alpha = 0.45) [41]. Subconstructs were identified within each broad construct, including four subconstructs within Structure (i.e., Meal and Snack Routines, Modeling, Rules and Limits, and Healthy Food Availability and Accessibility), four subconstructs within Coercive Control (i.e., Weight Concerns, Restriction, Pressure to Eat, and Threats and Bribes), and two subconstructs within Autonomy Support (i.e., Child Involvement: Planning Meals and Child Involvement: Shopping). (The final items, factor loadings, and internal consistency for each of Structure, Coercive Control, and Autonomy Support scales are presented in Supplementary Materials Tables S2–S4.)

### 2.3. Statistical Analysis

All data were prepared in Microsoft Access database files and were imported into Stata Statistical Software: Release 14 (StataCorp, 2015, College Station, TX, USA). If siblings were included in the study, one child was randomly chosen for the data analysis. All statistical analyses were conducted by Stata Statistical Software: Release 14 (StataCorp, 2015, College Station, TX, USA).

For parenting styles, chi-squared tests were used to examine differences in parents and children’ demographic information by parenting style.

For nutrition data, children’s average daily energy, macronutrient, selected micronutrient, and food group intakes were calculated from the HSFFQ. To examine the diet quality of the participants, nutrient density was used. Nutrient density was determined using the average of daily intake of nutrients per 1000 kcal intake. In addition, food group intakes were adjusted for energy intake (servings of food groups per 1000 kcal of total energy consumed). For example, if a participant consumed three servings of fruits and 2000 kcal of total energy intake per day, then the adjusted servings of fruits was 3/2000 * 1000 = 1.5 servings/1000 kcal per day.

For parenting practices, an average score for each of three constructs (i.e., Structure, Coercive Control, and Autonomy Support) and ten subconstructs of food parenting practices were calculated by averaging the contributing item scores. Kruskal–Wallis tests were performed to examine whether food parenting practices differed between the four parenting styles. Post hoc tests and Dunn’s tests were used to detail specific differences between the four types of parenting style [42,43]. Additionally, Spearman’s correlation analyses between children’s dietary intake (using nutrient density) and ten food parenting practices subconstructs were performed for statistical significance (*p* < 0.05). 

To examine the overall association between children’s dietary intake (using nutrient density) and parenting styles and food parenting practices, multiple linear regression analyses were performed. The distribution of dietary data did not follow a standard normal distribution. Therefore, linear models were bootstrapped with 500 repetitions to compute the SEs of the regression coefficient estimates from their empirical distributions. In each regression model, dependent variables were children’s daily food group intakes (i.e., fruit, vegetables, sweets, fats, dairy, grain, and protein), selected micronutrient intake (i.e., calcium, iron, zinc, vitamin C, vitamin B6, and folate), and macronutrient intake (i.e., total protein, total fats, and carbohydrates,), respectively. Independent variables are parenting practices constructs (i.e., Structure, Coercive Control, and Autonomy Support) and parenting styles. The authoritative style served as the referent parenting style, meaning that the authoritarian, indulgent, and uninvolved feeding styles were compared against the authoritative feeding style in the model. Models were adjusted for parent education level, child gender, and child BMI percentile category, so that the confounding effects could be reduced. A significance level of 0.05 was applied for all analysis.

## 3. Results

### 3.1. Participant Characteristics

Of the 293 recruited, 166 parent participants completed all the surveys. Table 1 shows the general characteristics for participants. The average age of parents was 34 ± 0.4 years. Parents in this study were predominately mothers (83.0%) and white (91.1%). Parents had BMIs considered normal/underweight (46.1%); the majority had a household income greater than $60,000 (78.1%) and a bachelor’s degree or higher (79.4%). The average age of children was 3.7 ± 0.1 years, and 47.6% were boys and 52.4% were girls. The majority were white (85.5%) and were in a normal/underweight BMI percentile (81.1%). Overall, 34.3% parents in this study had an authoritative parenting style, 34.3% were authoritarian, 17.5% were indulgent, and 13.9% were uninvolved. The chi-square test did not show any significant differences in parenting style nor in any of the measured demographic variables.

### 3.2. Food Parenting Practices by Parenting Styles

Table 2 shows food parenting practice scores by parenting styles. In this study, authoritarian parents have more Weight Concern practices than authoritative and indulgent parents. Authoritative parents have more Autonomy Support practices than the other three parenting styles. Uninvolved parents had more Weight Concern practices and fewer Autonomy Support practices (especially Child Involvement: Shopping practices) compared to authoritative parents.

### 3.3. Correlation between Children’s Daily Dietary Intakes and Food Parenting Practice Subconstructs

Table 3 shows the correlation analyses between children’s daily dietary intakes and food parenting practices. For children, most food parenting practices within Structure had positive relationships, with a healthy nutrient density and food group intakes (servings/1000 kcal) and negative relationships with unhealthy intakes. For example, the subconstruct of Meal and Snack Routines was positively related to the intake of iron (r = 0.177), folate (r = 0.214), and grains (r = 0.164), and was negatively related to the intake of sweets (r = −0.182) and fats (r = −0.162). The subconstruct of Modeling was positively related to the intake of carbohydrates (r = 0.26), folate (r = 0.203), and fruits (r = 0.200), and negatively related to the intake of fats (r = −0.328). The Rules and Limits subconstruct was positively related to the intake of carbohydrates (r = 0.153), iron (r = 0.181), vitamin C (r = 0.246), fruits (r = 0.157), and vegetables (r = 0.238), and was negatively related to the intake of fats (r = −0.162). The Healthy Food Availability and Accessibility subconstruct was positively related to the intake of iron (r = 0.273), vitamin B6 (r = 0.323), fruits (r = 0.192), vegetables (r = 0.283), and protein (r = 0.173), and was negatively related to the intake of fats (r = −0.183) and sweets (r = −0.231). However, Rules and Limits and Healthy Food Availability and Accessibility subconstructs were also found to have a negative relationship with the intake of calcium and dairy.

Food parenting practices within Coercive Control mostly had positive relationships with unhealthy children’s nutrient and food group intakes. For example, the Weight Concerns subconstruct was positively related to the intake of fats (r = 0.161) and sweets (r = 0.156). The Threats and Bribes subconstruct was positively related to the intake of sweets (r = 0.187) and fats (r = 0.166). In addition, the Coercive Control subconstruct mostly had negative relationships with healthy children’s nutrient and food group intakes. For example, the Weight Concerns subconstruct was negatively related to the intake of vitamin A (r = −0.170); The Restriction subconstruct was negatively related to the intake of dairy (r = −0.162); the Pressure to Eat subconstruct was negatively related to the intake of calcium (r = −0.168) and dairy (r = −0.162); and the Threats and Bribes subconstruct was negatively related to the intake of iron (r = −0.167). 

Food parenting practices within Autonomy Support mostly had positive relationships with healthy children’s nutrient and food group intakes. The Child Involvement: Shopping subconstruct was positively related to the intake of carbohydrates (r = 0.165), vitamin C (r = 0.205), vitamin B6 (r = 0.210), vitamin A (r = 0.230), fruits (r = 0.175), and vegetables (r = 0.264), and was negatively related to the intake of fats (r = −0.224).

In general, for children, food parenting practices within Structure and Autonomy Support were mostly positively related to the healthy daily dietary intakes and were negatively related to the unhealthy intakes. Food parenting practices within Coercive Control mostly had positive relationships with unhealthy dietary intakes and negative relationships with healthy dietary intakes.

### 3.4. Adjusted Regression Models for Children’s Dietary Intake

Table 4 reports adjusted regression models for children’s daily food group intake. The results show that children with authoritative parents consumed more fruits compared to children with authoritarian parents (coef. = −0.421, *p* = 0.008) and indulgent parents (coef. = −0.569, *p* = 0.001). Children with parents who have more Structure practices consumed more vegetables (coef. = 1.310, *p* = 0.018) and fewer sweets (coef. = −0.748, *p* = 0.012). Children with parents who have more Structure practices (coef. = −1.289, *p* = 0.007) and more Coercive Control practices (coef. = −1.364, *p* = 0.004) consumed less dairy. Children with parents who have more Autonomy Support practices consumed less grain (coef. = −1.031, *p* = 0.042). In addition, children with uninvolved parents (coef. = 0.304, *p* = 0.015) consumed more protein compared to children with authoritative parents.

The strong link between Autonomy Support and parenting style raises the issue of collinearity between these two variables and the relevance on including both of the same in one model. Regression models by dropping the Autonomy Support variable were conducted and the major findings were the same (Supplementary Materials Table S5). In addition, child’s BMI could be considered to be a consequence of parenting practices and dietary intake more than a cause of dietary intake. Adjusted regression models not on child’s BMI were also conducted. Compared to Table 4, the major findings are the same except for protein no longer being significant (Supplementary Materials Table S6).

Table 5 reports adjusted regression models for children’s daily micronutrient and macronutrient intake. The results showed that children with parents who have more Structure parenting practices consumed more iron (coef. = 1.693, *p* = 0.001) and folate (coef. = 68.521, *p* = 0.007), as well as less calcium (coef. = −242.015, *p* = 0.012) and total fats (coef. = −6.358, *p* = 0.050). Children with parents who have more Coercive Control parenting practices consumed more iron (coef. = 1.080, *p* = 0.029), vitamin B6 (coef. = 0.133, *p* = 0.045), and less calcium (coef. = −250.694, *p* = 0.007). 

## 4. Discussion

This study examined the relationship between parenting styles/food parenting practices and dietary intakes of preschool-aged children. In general, our findings showed that food parenting practices had a higher number of specific significant findings on children’s nutrient and food group intakes than parenting styles. Positive parenting practices within Structure were significantly related to healthier children’s dietary intakes (e.g., vegetables, iron, and folate) and less unhealthy dietary intakes (e.g., sweets and total fats). Children with authoritative parents consumed more fruits compared to children with authoritarian parents and indulgent parents.

For food parenting practices and preschoolers’ dietary intakes, our findings from Spearman’s correlation analysis confirmed the results of previous studies that more nondirective child-centered food practices (such as Structure and Autonomy Support constructs) were related to consuming healthier diets (e.g., fruit and vegetable consumption) [44,45,46], and Coercive Control practices were associated with unhealthy behaviors (e.g., sweets and fats consumption) [47]. Our study also provided more associations between specific food parenting practices and children’s energy and nutrient intakes. For example, Meal and Snack Routines were associated with a higher consumption of iron and folate; Rules and Limits, Healthy Food Availability and Accessibility, and Child Involvement: Shopping were associated with higher consuming of vitamin C, B6, A, and folate. These findings were consistent with the similar associations reported in our study, in which these food parenting practices were also related to a higher consumption of food groups which contains these nutrients (e.g., grain, fruits, and vegetables).

The associations between food parenting practices and children’s dietary intakes did produce some unexpected results. For example, parenting Structure practices (e.g., Rules and Limits and Healthy Food Availability and Accessibility) were found to be negatively related to dairy intake; Coercive Control practices (e.g., Pressure to Eat) had a weak positive relationship with vegetable intakes; and Autonomy Support practices (e.g., Child Involvement: Planning Meals) had a weak negative relationship with grain intake. The distinction between coercive and noncoercive types of restriction may help with a better understanding of Structure and Coercive Control practices. Previous studies examining how Structure practices—such as Rules and Limits—influence child eating behaviors show inconsistent findings [24]. While some studies suggest that Rules and Limits are associated with higher-intake healthier diets [48,49], others show different associations between unhealthy foods [50,51]. This could be because even though Rules and Limits focus on a noncoercive practice, it still has the property in enforcing parent-centered limits on a child’s access of foods. Regarding the Pressure to Eat, it is important to distinguish between practices that pressure a child to eat more food and those that pressure a child to eat more healthy foods [24]. Intake of vegetables may be more strongly related to the use of pressure to eat healthy foods [24]. Additionally, food parenting practices together determine the home food environment. The home environment that parents create through one parenting practice is also likely to influence the use of other practices. Thus, it is important to understand how practices are used in combination and how they might influence child dietary behaviors in the future.

When considering the influences of parenting styles and food parenting practices together, our findings from adjusted regression models showed that food parenting practices had a higher number of specific significant findings in terms of children’s dietary intakes than parenting styles. However, unexpected results were found, including positive parenting practices related to less dairy and grain intake, and uninvolved parents related to more protein intake. One possible reason for this is because food groups determined by the HSFFQ in this study contained some unhealthy foods. For example, food group dairy not only had milk, cheese, and yogurt, but also had pizza and pudding. The grain food group contained all grains and was not limited only to whole grain foods. Food group protein contained foods such as hamburger and hotdog. The present classification probably leads to an overestimation of food group intake for some individuals and the unexpected associations between feeding practices and dietary intake.

No significant differences were seen in this study for most assessed children’s dietary intakes between authoritative parenting style and the other three parenting styles, except for fruits. The results found here were consistent with our hypothesis and previous research, which found fruit consumption to be higher in children with authoritative parents [9,52]. A system review reported that an authoritative parenting style is not only important for a healthier dietary intake, but is also important for better child outcomes (e.g., school achievement and social adjustment) [53]. Therefore, future interventions could work toward modifying or achieving an authoritative parenting style, as parenting styles have been shown to be malleable [54].

Limitations to this study are important to acknowledge. Parent-reported data were used to assess parenting style and dietary intake. A food frequency survey was used to assess the intake of nutrients and food groups. Overreporting or underreporting may exist and can affect the usefulness of the collected dietary data [55]. Food parenting practices were measured using CHES. Low alpha of the Autonomy Support scale (0.45) is another limitation. In addition, the sample population in this study was limited to 1) children (one child from one parent) who attended daycare centers or preschool; 2) parents who mainly were mothers and had higher household income and education levels compared to the state population [56]. This may limit the generalizability to different age groups of children or a larger level population. In addition, the analyses of this research were based on a cross-sectional study. The results only present the relationships between food parenting practices/parenting styles and children’s dietary intakes, and causality is difficult to establish. Longitudinal studies are needed in the future to examine the long-term impacts of food parenting practices and parenting styles on children’s diet. Finally, this paper tested a large set of hypotheses between each nutrient/food group and each parenting style or practice; however, no correction for multiple testing false discovery rates were conducted. This resulted in an increased risk of a false-positive finding, which potentially increased the probability of observing at least one significant result just due to chance.

One of the study strengths is the age group assessed, as preschool age is an important developmental period for adopting healthy eating habits. In addition, this study provides comprehensive information on dietary intakes, including energy intake, nutrient intake, and food group intake.

## 5. Conclusions

With the present study, we used data from a sample of preschoolers to investigate the relationship between parenting styles, food parenting practices, and dietary intakes of selected nutrients and food groups. The results addressed the importance of parental influences for preschoolers’ healthy dietary intakes, which suggested that future interventions and educational programs could enhance parenting practices to impact child diet and eating behaviors. Additionally, to establish a causal relationship and to examine the long-term impacts of parents, the processes of how parents impact their children’s dietary intakes still needs more investigation work. Furthermore, future research can apply for a larger sample or population.

## Figures and Tables

**Table 1:** Frequency of parents and children demographic information according to parenting style.

Variable, N ^1^ (%)	Overall ^2^	Authoritative ^3^	Authoritarian ^3^	Indulgent ^3^	Uninvolved ^3^	*p* Value ^4^
Overall Sample	166 (100)	57 (34.34)	57 (34.34)	29 (17.47)	23 (13.86)	
*Parent Gender Role:*						0.282
Mother	137 (83.03)	49 (35.77)	46 (33.58)	21(15.33)	21(15.33)	
Father	28 (16.97)	8 (28.57)	10 (35.71)	8 (28.57)	2 (7.14)	
*Parent BMI:*						0.675
Normal/underweight	76 (46.06)	28 (36.84)	25 (32.89)	15 (19.74)	8 (10.53)	
Overweight/obese	89 (53.94)	29 (32.58)	32 (35.96)	14 (15.73)	14 (15.73)	
*Parent Race:*						0.186
White	144 (91.14)	52 (36.11)	48 (33.33)	25 (17.36)	19 (13.19)	
Non-white	14 (8.86)	2 (14.29)	4 (28.57)	4 (28.57)	4 (28.57)	
*Household Income:*						0.995
Less than $60,000	36 (21.95)	13 (36.11)	12 (33.33)	6 (16.67)	5 (13.89)	
$60,000 or greater	128 (78.05)	44 (34.38)	44 (34.38)	23 (17.97)	17 (13.28)	
*Parent Education:*						0.361
Associate’s degree or less	34 (20.61)	10 (29.41)	16 (47.06)	5 (14.71)	3 (8.82)	
Bachelor’s degree or higher	131 (79.39)	46 (35.11)	41 (31.3)	24 (18.32)	20 (15.27)	
*Child Gender:*						
Boy	79 (47.59)	22 (27.85)	29 (36.71)	13 (16.46)	15 (18.99)	0.168
Girl	87 (52.41)	35 (40.23)	28 (32.18)	16 (18.39)	8 (9.2)	
*Child Race:*						0.180
White	142 (85.54)	52 (36.62)	50 (35.21)	22 (15.49)	18 (12.68)	
Non-white	24 (14.46)	5 (20.83)	7 (29.17)	7 (29.17)	5 (20.83)	
*Child BMI Percentile:*						0.496
Normal/underweight	133 (81.10)	49 (36.84)	44 (33.08)	21 (15.79)	19 (14.29)	
Overweight/obese	31 (18.90)	8 (25.81)	11 (35.48)	8 (25.81)	4 (12.9)	

^1.^ Not all numbers may add up due to parents skipping. ^2.^ % = column percentage. ^3.^ % = row percentage. ^4.^ Chi-square test.

**Table 2:** Summary of food parenting practices by four parenting styles (*n* = 166).

Parenting Practices	Authoritative (At)		Authoritarian (Ar)		Indulgent (In)		Uninvolved (Un)		Total		Kruskal–Wallis Test (*p* Value)	post hoc Test: Dunn’s Test
	Mean	SD	Mean	SD	Mean	SD	Mean	SD	Mean	SD		
Structure:	0.67	0.12	0.64	0.11	0.63	0.17	0.63	0.12	0.65	0.13	0.105	
1. Meal and Snack Routines	0.82	0.19	0.80	0.17	0.78	0.20	0.75	0.17	0.80	0.18	0.352	
2. Modeling	0.71	0.13	0.69	0.11	0.67	0.16	0.70	0.11	0.70	0.13	0.304	
3. Rules and Limits	0.48	0.25	0.42	0.24	0.43	0.30	0.41	0.27	0.44	0.26	0.450	
4. Healthy Food Availability and Accessibility	0.68	0.11	0.62	0.13	0.61	0.17	0.61	0.14	0.64	0.13	0.065	
Coercive Control:	0.36	0.14	0.36	0.14	0.35	0.15	0.36	0.12	0.36	0.14	0.882	
5. Weight Concerns	0.11	0.21	0.17	0.18	0.14	0.24	0.15	0.14	0.14	0.20	0.023 *	At < Ar *, Ar > In *, At < Un *
6. Restriction	0.58	0.24	0.59	0.28	0.55	0.25	0.55	0.25	0.58	0.25	0.703	
7. Pressure to Eat	0.44	0.27	0.39	0.22	0.43	0.27	0.45	0.23	0.42	0.25	0.758	
8. Threats and Bribes	0.24	0.12	0.24	0.14	0.20	0.13	0.25	0.13	0.24	0.13	0.708	
Autonomy Support:	0.65	0.10	0.56	0.10	0.61	0.11	0.56	0.10	0.60	0.11	0.000 *	At > Ar *, At > In *, At > Un *
9. Child Involvement: Planning Meals	0.54	0.16	0.47	0.15	0.49	0.17	0.45	0.18	0.50	0.16	0.071	
10. Child Involvement: Shopping	0.72	0.13	0.62	0.11	0.68	0.15	0.64	0.09	0.67	0.13	0.001 *	At > Ar *, Ar < In *, At > Un *

Note: * *p* < 0.05. The scale for parenting practices ranges from 0–1. For Structure, higher responding scores reflect the greater use of positive structure practices. For Coercive Control, which includes negative behaviors, higher responding scores reflect the greater use of those practices. For Autonomy Support, higher responding scores reflect greater child control for positive practices, while lower scores reflect a greater parent control.

**Table 3:** Spearman’s correlation analysis between daily dietary intakes and food parenting practices subconstructs for children.

Children’s Daily Dietary Intakes/1000 kcal	Food Parenting Practices									
	Structure				Coercive Control				Autonomy Support	
	Meal and Snack Routines	Modeling	Rules and Limits	Healthy Food Availability and Accessibility	Weight Concerns	Restriction	Pressure to Eat	Threats and Bribes	Child Involvement: Planning Meals	Child Involvement: Shopping
Energy Density (g/1000 kcal):										
Protein	0.071	0.049	-0.078	0.061	0.019	−0.053	−0.079	−0.079	−0.002	0.045
Fats	−0.105	−0.318 *	−0.162 *	−0.183 *	0.161 *	−0.090	−0.139	0.117	0.001	−0.224 *
Carbohydrates	0.049	0.260 *	0.153 *	0.118	−0.144	0.087	0.137	−0.057	0.021	0.165 *
Selected Nutrient Density ^1^:										
Calcium	−0.006	−0.097	−0.190 *	−0.188 *	−0.144	−0.131	−0.168 *	0.076	−0.038	0.006
Iron	0.177 *	0.135	0.181 *	0.273 *	−0.102	0.126	0.166 *	−0.167 *	−0.028	0.093
Zinc	0.095	−0.102	−0.006	0.115	−0.046	−0.046	−0.023	−0.073	0.020	0.054
Vitamin C	0.042	0.076	0.246 *	0.125	−0.018	0.099	0.150	−0.075	0.079	0.205 *
Vitamin B6	0.136	0.123	0.126	0.323 *	0.025	0.081	0.087	−0.108	0.095	0.210 *
Vitamin A	0.030	0.033	0.057	0.093	−0.170 *	−0.067	0.027	−0.013	0.055	0.230 *
Folate	0.214 *	0.203 *	0.134	0.143	−0.080	0.046	0.142	−0.151	−0.002	0.096
Food Group Intake (servings/1000 kcal):										
Dairy	−0.039	−0.120	−0.200 *	−0.232 *	−0.124	−0.162 *	−0.162 *	0.090	−0.019	−0.041
Fruits	0.049	0.200 *	0.157 *	0.192 *	−0.019	0.108	−0.030	−0.098	0.096	0.175 *
Vegetables	0.131	0.081	0.238 *	0.283 *	−0.053	0.029	0.178 *	−0.023	0.097	0.264 *
Grains	0.164 *	0.048	0.022	0.037	−0.086	0.087	0.093	−0.080	−0.168 *	−0.088
Protein	0.131	0.010	0.078	0.173 *	0.076	0.045	−0.015	−0.089	0.017	0.061
Sweets	−0.182 *	−0.147	−0.031	−0.231 *	0.156 *	0.078	0.007	0.187 *	0.073	−0.097
Fats	−0.162 *	−0.272 *	−0.012	−0.051	0.129	0.004	0.051	0.166 *	0.012	−0.098

Note: * *p* < 0.05. ^1.^ Units for nutrients: calcium (mg/1000 kcal), iron (mg/1000 kcal), zinc (mg/1000 kcal), vitamin C (mg/1000 kcal), vitamin B6 (mg/1000 kcal), vitamin A (mcg/1000 kcal), and folate (mcg/1000 kcal).

**Table 4:** Adjusted regression models for children’s daily food group intake.

Variables	Children’ Daily Food Group Intake (servings/1000 kcal)													
	Fruits		Vegetables		Sweets		Fats		Dairy		Grain		Protein	
	Coef. ^1^	*p* > |z|	Coef.	*p* > |z|	Coef.	*p* > |z|	Coef.	*p* > |z|	Coef.	*p* > |z|	Coef.	*p* > |z|	Coef.	*p* > |z|
Model ^2^	P = 0.0248 * R^2^ = 0.1441		P = 0.0004 * R^2^ = 0.1406		P = 0.0001 * R^2^ = 0.1576		P = 0.0506 R^2^ = 0.0822		P = 0.0317 * R^2^ = 0.1037		P = 0.0002 * R^2^ = 0.1341		P = 0.0272 * R^2^ = 0.1209	
Parenting Practices:														
Structure	0.727	0.168	1.310	0.018 *	−0.748	0.012 *	−0.721	0.047	−1.289	0.007 *	0.610	0.116	0.594	0.058
Coercive Control	0.300	0.515	0.702	0.110	0.361	0.184	0.336	0.285	−1.364	0.004 *	0.119	0.708	0.309	0.221
Autonomy Support	1.045	0.170	0.772	0.187	0.375	0.289	−0.217	0.662	0.150	0.837	−1.031	0.042 *	0.059	0.880
Parenting Styles ^3^:														
Authoritarian	−0.421	0.008 *	−0.144	0.317	0.156	0.082	0.074	0.552	0.015	0.930	−0.000	1.000	0.098	0.217
Indulgent	−0.569	0.001 *	0.221	0.220	−0.136	0.175	−0.105	0.404	0.118	0.533	0.054	0.644	0.185	0.084
Uninvolved	−0.291	0.188	0.248	0.216	−0.031	0.786	0.055	0.709	−0.059	0.793	−0.083	0.576	0.304	0.015 *

Note: * *p* < 0.05. ^1.^ Coef. = observed coefficients. ^2.^ Results from separate bootstrapping regression models for each independent variable, adjusted for parent education level, child gender, and child BMI percentile category. ^3.^ The authoritative style served as the referent parenting style, meaning that the authoritarian, indulgent, and uninvolved feeding styles were compared against the authoritative feeding style in the model.

**Table 5:** Adjusted regression models for children’s daily nutrient intake.

Variables	Children’ Daily Nutrient Intake/1000 kcal ^1^											
	Calcium		Iron		Vitamin C		Vitamin B6		Folate		Total Fats	
	Coef. ^2^	P > |z|	Coef.	P > |z|	Coef.	P > |z|	Coef.	P > |z|	Coef.	P > |z|	Coef.	P > |z|
Model ^3,4^	P = 0.0469 * R^2^ = 0.0945		P = 0.0002 * R^2^ = 0.1811		P = 0.0168 * R^2^ = 0.1179		P = 0.0009 * R^2^ = 0.1480		P = 0.0032 * R^2^ = 0.1167		P = 0.0498 * R^2^ = 0.1068	
Parenting Practices:												
Structure	−242.015	0.012 *	1.693	0.001 *	17.824	0.193	0.143	0.067	68.521	0.007 *	−6.358	0.050 *
Coercive Control	−250.694	0.007 *	1.080	0.029 *	19.285	0.182	0.133	0.045 *	26.910	0.222	−2.640	0.326
Autonomy Support	57.368	0.673	−0.344	0.586	21.557	0.188	0.130	0.135	9.294	0.761	−1.764	0.644
Parenting Styles ^5^:												
Authoritarian	2.009	0.949	0.036	0.823	−8.682	0.071	−0.036	0.104	4.700	0.546	1.668	0.051
Indulgent	27.853	0.434	0.358	0.081	−7.141	0.142	0.008	0.783	15.253	0.081	1.434	0.134
Uninvolved	−11.970	0.773	0.192	0.329	−3.366	0.481	−0.017	0.543	15.937	0.096	1.684	0.088

Note: * *p* < 0.05. ^1.^ Units for nutrients: calcium (mg/1000 kcal), iron (mg/1000 kcal), zinc (mg/1000 kcal), vitamin C (mg/1000 kcal), vitamin B6 (mg/1000 kcal), vitamin A (mcg/1000 kcal), folate (mcg/1000 kcal), and total fats (g/1000 kcal). ^2.^ Coef. = observed coefficients. ^3.^ Results from separate bootstrapping regression models for each independent variable, adjusted for parent education level, child gender, and child BMI percentile category. ^4.^ Adjusted regression models for children’s daily intakes of zinc (P = 0.4873, R^2^ = 0.0568), vitamin A (P = 0.0655, R^2^ = 0.0863), total protein (P = 0.4182, R^2^ = 0.0610), and carbohydrates (P = 0.2237, R^2^ = 0.0833) were not significant. These results were not presented in the table. ^5.^ The authoritative style served as the referent parenting style, meaning that the authoritarian, indulgent, and uninvolved feeding styles were compared against the authoritative feeding style in the model.

## Data Availability

The data presented in this study are available on request from the corresponding author.

## Supplementary Materials

The following are available online at https://www.mdpi.com/article/10.3390/nu13103630/s1, File S1: Step to step instructions for HSFFQ analysis, Table S1: The dimensions and items of the PDI-S survey used in this study, Table S2: Food parenting practices exploratory factor analysis for Structure, Table S3: Food parenting practices exploratory factor analysis for Coercive Control, Table S4: Food parenting practices exploratory factor analysis for Autonomy Support, Table S5: Multiple regression models for children’s daily food group intake (without Autonomy Support variable), Table S6: Multiple regression models for children’s daily food group intake (not adjusted on child’s BMI percentile category).
