# Supplementary material for: Parenting Styles, Food Parenting Practices and Dietary Intakes of Preschoolers
Source: Nutrients. 2021 Oct 16;13(10):3630. doi: 10.3390/nu13103630 (PMC8537258; doi:10.3390/nu13103630)
Supplement: Supplementary file 1 [file nutrients-13-03630-s001.zip › nutrients-1385684-supplementary materials-1.pdf]

File S1. Step to step instructions for HSFFQ analysis.

1) Convert food frequencies to daily servings by using "88GP frequency factors" download from HSFFQ website (Figure A).[1] For example, if a child participant stated eating an apple a day, the mean servings per day would be 1.0. Food groups (meat, bread/cereal, milk, vegetables, fruit, sweets and fats) were determined by the HSFFQ (Figure B) (*The HSFFQ User's Manual* Page F-8).[2] The servings of food items from the same group were added to the sum of "Mean Servings per Day Reported by Food Group".

2) Convert food frequencies to food amount (g/day) for each food item. *The HSFFQ User's Manual Page E-48* provided portion size in gram weight assigned to each food item on the HSFFQ (Figure C).[2] Portion size assigned weights of women were used for adult participants, and portion size assigned weights of 3-5 years children were used for child participants. Then food weight in gram for each food item was calculated by multiplying "daily number of servings to each food item" by "portion size in gram weight assigned to foods". For example, if a child participant stated eating an apple a day, the mean servings per day would be 1.0, the portion size in gram weight assigned to apple from Figure C was 124 g/serving, then the mean food weight per day to apple would be 1.0 * 124 = 124g.

For the food items on the HSFFQ whose the portion sizes were not given in the Figure C, using "What's In the Foods You Eat" search tool[3] to find measure weights for one serving size directly. These food items include "Fruit cocktail", "Zucchini", "Popcorn or pretzels", "Beer, wine, wine cooler, mixed drink or liquor", "Tacos, burritos", "Fried Chicken, chicken nuggets", "Other chicken or turkey", "Butter (not margarine)" and "Margarine". The final portion size in gram weight assigned to each food item on the HSFFQ used in this study were presented in Table D. In this way we were able to calculate amounts of food consumption per day for participants of this study.

3) Nutrient data per 100 gm portion for the food items were derived from the HSFFQ and estimated using the Harvard nutrient database.[1] Table E presents the nutrient data for food items on the HSFFQ used in this study. Daily nutrients intakes from the HSFFQ were computed by converting the food frequency to a daily number of servings to each food item, multiplying this by the nutrient content for the assigned weight, and summing the value for all foods. The current version of the HSFFQ estimates seven micronutrients: calcium, iron, zinc, vitamin C, vitamin B6, vitamin A, and folate. Total calories, protein, carbohydrates, and fats intakes were calculated in the same way. Since carbohydrates and protein each provide 4 calories per gram, and fat contains 9 calories in each gram, then daily calorie sources (protein, carbohydrates, and fats) were reported.

The HSFFQ provided dietary intakes results regarding: 1) daily nutrient intakes (vitamin A, vitamin C, calcium, zinc, folate, iron, protein, and vitamin B6); 2) daily calorie intakes and sources (carbohydrates, protein, fats); 3) daily mean servings by food groups (meat, bread/cereal, milk, vegetables, fruit, sweets and fats).

Result 3 "mean servings by food group" was a direct reporting of the number of servings that the participants reported. The Result 1 "selected nutrient intakes" and Result 2 "calorie intakes and sources" were calculated from the frequency reported and the nutrients in the corresponding serving of food.[2]

References:

1. Harvard T.H. Chan School of Public Health Nutrition Department's file download site. Available online: https://regepi.bwh.harvard.edu/health/nutrition.html (accessed on 11 December 2020).
2. Colditz, G. Dietary intake and health outcomes: final report. U.S. Department of Agriculture, Economic Research Service. Available online: https://naldc.nal.usda.gov/download/32818/PDF (accessed on 11 December 2020).
3. What's in the foods you eat search tool. U.S. Department of Agriculture website. Available online: https://reedir.arsnet.usda.gov/codesearchwebapp/(S(z2vxwy1lgtfuvqjyz43troym))/CodeSearch.aspx (accessed on 11 December 2020).

How do I calculate the number of servings of fruits or vegetables per day?

If you would like to calculate the number of servings of any food grouping, you must sum the daily frequencies reported for the foods you select to represent your group. The frequency weights for the 88GP, 80outGP and the 97GP are listed below.

Example: 88GP servings of fruit per day
Participant reports:
Raisins 1-3 per month
Apple 1 per day
Orange 2-4 per week
Other fruit juices 2-3 times per day

Frequency factors:
Raisins – 0.08
Apple – 1.0
Oranges – 0.43
Other fruit juices – 2.5

Sum frequency factors: 0.08 + 1.0 + 0.08 + 2.5 = 3.66 servings per day of fruits
******************88GP frequency factors: ************************

ffwgt1	0; never (default value for no answer)
ffwgt2	0.08; 1-3/mo
ffwgt3	0.14; 1/wk
ffwgt4	0.43; 2-4/wk
ffwgt5	0.8; 5-6/wk
ffwgt6	1.0; 1/day
ffwgt7	2.5; 2-3/day
ffwgt8	4.5; 4-5/day
ffwgt9	6.0; 6/day

Figure A. How do I calculate the number of servings of fruits or vegetables per day?

Source: Harvard T.H. Chan School of Public Health Nutrition Department's file download site. Available at: https://regepi.bwh.harvard.edu/health/nutrition.html (accessed on 11 December 2020).

meat=hamburg, hotdog, bologna, tuna, baked beans, other bean, liver, fr chicken, other chicken, pork, beef, fr fish, other fish and egg.

brdcer=crackers, rice, spaghetti, pizza, macaroni and cheese, bread, hot cereal, cold cereal, pancakes, english muffin, biscuits and cornbread.

milks=milk, cheese, pizza, pudding, yogurt, and macaroni and cheese.

veg=corn, peas, tomato, peppers, carrots, broccoli, green beans, spinach, greens, mixed vegetables, squash, zucchini, french fries, potatoes, sweet potatoes, coleslaw, okra, brussel, sprouts, lettuce, salad, and vegetable soup.

fruit=wic juice, oj, juice, orange, banana, apple, grapes, peaches, strawberr, melon, watermelon, pineapple, raisins,&apricots

vitc=oj, orange, strawberries, melon, and broccoli.

vita=melon, carrots, spinach, greens, squash, sweet potatoes, liver, and pumpkin pie.

sweet=hot choc, pudding, ice cream, fruit drinks, cookies, cake, pies, jello, chocolate, candy, sweet roll, pancake, donut, and soda.

fats=ice cream, cheese, mayo, chips, cookies, cake, pies, chocolate, coldcut, nuts, pb, bu, marg, fr chicken, fr fish, sausage, bacon ,donut, hotdogs and sweet roll.

Figure B. Food groups determined by the HSFFQ.

Source: *The HSFFQ User's Manual* Page F-8: Colditz G. Dietary intake and health outcomes: final report. U.S. Department of Agriculture, Economic Research Service. Available at: https://naldc.nal.usda.gov/download/32818/PDF (accessed on 11 December 2020).

TABLE 2
FOOD PORTION SIZE IN GRAM WEIGHT ASSIGNED TO FOODS ON THE HSFFQ

Food Name	Portion Size		
	Women	Children 1-2 years	Children 3-5 years
Milk	270	183	183
Hot Chocolate	28	28	28
Cheese, plain, in sandwiches	28	21	21
Yogurt	198	117	117
Ice Cream	90	67	67
Pudding	119	80	120
Orange	122	131	131
Orange juice	217	124	155
Apple juice	255	186	186
Kool Aid, other fruit drinks	270	124	155
Banana	102	91	102
Apple or applesauce	138	105	124
Grapes	75	75	75
Peaches	166	123	149
Strawberries	47	47	47
Cantaloupe	127	68	68
Watermelon	440	120	120
Pineapple	80	47	47
Raisins	28	14	28
Corn	83	42	64
Peas	52	42	42
Tomatoes	62	31	31
Peppers	18	9	9
Carrots	70	29	29
Broccoli	70	46	46
Green beans	40	35	35
Spinach	70	27	27
Greens	72	27	27
Squash, orange or winter	156	49	60
French fries, fried potatoes	115	42	51
Potatoes	99	72	80
Sweet potatoes or yams	70	49	60
Cabbage or coleslaw	67	33	41
Lettuce salad	55	37	37
Salad dressing or mayonnaise	15	5	12
Chips (potato, corn, others)	42	13	20
Nuts	15	15	15
Cookies or brownies	25	22	22
Cake or cupcake	66	30	30
Pie (pumpkin,etc.)	140	37	37
Other pie	112	66	66
Jello	90	90	90
Chocolate candy	32	18	27
Other candy	15	18	18
Tea	180	120	120
Soft drinks	246	124	186
Sugar-free soft drinks	246	124	186
Baked beans or chili beans	128	83	128
Other dried beans, peas	85	58	58
Rice	113	87	87
Spaghetti or other pasta	310	192	227
Pizza	140	72	77
Macaroni and cheese	168	121	168

Figure C. Portion size in gram weight assigned to each food item on the HSFFQ.

TABLE 2
FOOD PORTION SIZE IN GRAM WEIGHT ASSIGNED TO FOODS ON THE HSFFQ

Food Name	Portion Size Women	Children 1-2 years	Children 3-5 years
Hot dogs	44	44	44
Hamburgers	121	72	105
Canned tuna	60	49	49
Cold cuts	28	28	28
Peanut butter	16	16	16
Bread, toast, or rolls	25	25	25
Pork chops	84	39	56
Steak or roast beef	108	56	56
Fish	85	48	56
Liver	84	42	42
Sausage	54	28	28
Bacon	16	16	16
Hot cereal or grits	180	154	234
Cold breakfast cereal	28	21	30
Doughnut	43	38	43
Sweet roll or muffin	57	44	58
Pancake	33	33	33
English muffin or bagel	50	25	25
Biscuit	28	25	32
Cornbread or tortillas	56	51	56
Vegetable soup	270	122	244
Other soup	217	122	241
Crackers	15	12	12
Eggs	46	59	66
Mixed vegetables	116	89	69

Figure C *continue*. Portion size in gram weight assigned to each food item on the HSFFQ.

Source: *The HSFFQ User's Manual* Page E-48: Colditz G. Dietary intake and health outcomes: final report. U.S. Department of Agriculture, Economic Research Service. Available at: https://naldc.nal.usda.gov/download/32818/PDF (accessed on 11 December 2020).

Table D. Portion size in gram weight assigned to each food item on the HSFFQ.

Food item on the HSFFQ	Portion size in gram weight assigned to food item	
	Children 3-5 years	Adult
Milk	183	270
Hot Chocolate	28	28
Cheese, plain or in sandwiches	21	28
Yogurt	117	198
Ice cream (cones, sandwiches, sundaes)	67	90
Pudding	120	119
Orange juice or grapefruit juice	155	217
Other juice	186	255
Fruit drinks (Hi-C, Kool-aid, lemonade, sportsdrink)	155	270
Banana	102	102
Peaches	149	166
*Fruit cocktail, mixed fruit	117	149
Orange or Grapefruit	131	122
Apple or pear	124	138
Applesauce	124	138
Grapes	75	75
Strawberries	47	47
Melon (Cantaloupe)	68	127
Pineapple	47	80
Raisins or prunes	28	28
Corn	64	83
Peas	42	52
Tomatoes, tomato sauce, salsa	31	62
Peppers (green, red, hot)	9	18
Carrots	29	70
Broccoli	46	70
Green beans	35	40
Spinach	27	70
Greens(mustard,turnip,kale)	27	72
Mixed vegetables	69	116
Squash, orange or winter	60	156
*Zucchini, yellow squash	60	156
French Fries, fried potatoes	51	115
Potato (baked, boiled, or mashed)	80	99
Sweet potatoes or yams	60	70
Cabbage, coleslaw or cauliflower	41	67
Lettuce salad	37	55
Salad dressing	12	15
Mayonnaise	12	15
Chips (potato, corn, others)	20	42
*Popcorn or pretzels	20	42
Crackers	12	15
Nuts	15	15
Cookies or brownies	22	25
Cake or cupcake	30	66
Pie	37	140
Jello	90	90
Chocolate or candy bar	27	32
Other candy (not chocolate)	18	15
Coffee or tea	120	180
Soda, soft drink, pop (not sugar free)	186	246
Soda, soft drink, pop (sugar free)	186	246

Food item on the HSFFQ	Portion size in gram weight assigned to food item	
	**Children 3-5 years**	**Adult**
Beer,wine,wine cooler, mixed drink or liquor	.	116
Beans(baked, chili, or other)	128	128
Rice	87	113
Spaghetti or other pasta	227	310
Pizza	77	140
*Tacos, burritos	77	140
Macaroni and cheese	168	168
Hot dog	44	44
Sausage	28	54
Hamburger (prepared any way)	105	121
Canned Tuna	49	60
Fried fish, fish sticks	56	85
Other fish	56	85
Cold Cuts (baloney, ham, salami)	28	28
*Fried Chicken, chicken nuggets	56	85
*Other chicken or turkey	56	108
Pork or ham	56	84
Roast beef or steak	56	108
Liver, organ meats	42	84
Peanut butter	16	16
Bread (slice) toast, roll, or pita	25	25
*Butter (not margarine)	7	7
*Margarine	7	7
Vegetable Soup	244	270
Other soup	241	217
Cornbread or tortillas	56	56
Eggs	66	46
Bacon	16	16
Hot cereal or grits	234	180
Cold breakfast Cereal	30	28
Donut, fry bread	43	43
Sweet roll or muffin	58	57
Pancake, waffle, or French toast	33	33
English muffin or bagel	25	50
Biscuit	32	28

Note: Food items with * means whose the portion sizes were not given in the original *HSFFQ User's Manual*, then using "What's In the Foods You Eat" search tool to find measure weights for one serving size directly.

Table E. Nutrient data for food items on the HSFFQ used in this study.

Food item	Amount (g)	Total Calories (kcal)	Protein (gm)	Total Fat (gm)	Carbs (gm)	Calcium (mg)	Iron (mg)	Zinc (mg)	Vitamin C (mg)	Vitamin B6 (mg)	Vitamin A (mcg)	Folate (mcg)
Milk	100	61	3.2	3.2	4.8	113	0.03	0.37	0	0.04	46	5
Hot Chocolate	100	398	6.7	9.7	83.7	66.5	1.19	1.46	0.2	0.11	1	6
Cheese, plain or in sandwiches	100	368.5	18.1	31.2	4.2	1045	0.63	2.49	0	0.05	283.6	8
Yogurt	100	102	4.4	1.1	19	210	0.07	0.74	0.7	0.04	61.8	9
Ice cream (cones, sandwiches, sundaes)	100	207	3.5	11	23.6	128	0.09	0.69	0.6	0.05	117.6	5
Pudding	100	122.4	2	3.6	21.4	102	0.37	0.31	0	0.02	0	3
Orange juice or grapefruit juice	100	45	0.7	0.1	10.8	9	0.1	0.05	38.9	0.04	5	44
Other juice	100	46	0.1	0.1	11.3	8	0.12	0.02	0.9	0.02	0	0
Fruit drinks (Hi-C, Kool-aid, lemonade, sportsdrink)	100	35	0	0	9	16	0.05	0.02	11.2	0	0	0
Banana	100	89	1.1	0.3	22.8	5	0.26	0.15	8.7	0.37	3	20
Peaches	100	74	0.4	0.1	19.9	3	0.27	0.09	2.8	0.02	17	3
Fruit cocktail, mixed fruit	100	73	0.4	0.1	18.9	6	0.29	0.08	1.9	0.05	10	3
Orange or Grapefruit	100	47	0.9	0.1	11.8	40	0.1	0.07	53.2	0.06	11	30
Apple or pear	100	52	0.3	0.2	13.8	6	0.12	0.04	4.6	0.04	3	3
Applesauce	100	68	0.2	0.2	17.5	3	0.12	0.03	1.7	0.03	0.3	1
Grapes	100	69	0.7	0.2	18.1	10	0.36	0.07	3.2	0.09	3	2
Strawberries	100	32	0.7	0.3	7.7	16	0.41	0.14	58.8	0.05	1	24
Melon (Cantaloupe)	100	34	0.8	0.2	8.2	9	0.21	0.18	36.7	0.07	169	21
Pineapple	100	78	0.3	0.1	20.2	14	0.38	0.12	7.4	0.07	1	5
Raisins or prunes	100	299	3.1	0.5	79.2	50	1.88	0.22	2.3	0.17	0	5
Corn	100	81	2.5	0.7	19.3	3	0.47	0.63	3.5	0.1	10	35
Peas	100	78	5.2	0.3	14.3	24	1.52	0.67	9.9	0.11	105	59
Tomatoes, tomato sauce, salsa	100	18	0.9	0.2	3.9	10	0.27	0.17	13.7	0.08	42	15
Peppers (green, red, hot)	100	20	0.9	0.2	4.6	10	0.34	0.13	80.4	0.22	18	10
Carrots	100	35	0.8	0.2	8.2	30	0.34	0.2	3.6	0.15	852	14
Broccoli	100	35	2.4	0.4	7.2	40	0.67	0.45	64.9	0.2	77	108
Green beans	100	28	1.5	0.2	6.4	42	0.66	0.24	4.1	0.06	28	23
Spinach	100	23	3	0.3	3.8	136	3.57	0.76	9.8	0.24	524	146
Greens (mustard,turnip,kale)	100	28	1.9	0.4	5.6	72	0.9	0.24	41	0.14	681	13
Mixed vegetables	100	65	2.9	0.2	13.1	25	0.82	0.49	3.2	0.07	214	19
Squash, orange or winter	100	37	0.9	0.3	8.9	22	0.44	0.22	9.6	0.16	349.4	20
Zucchini, yellow squash	100	15	1.1	0.4	2.7	18	0.37	0.33	12.9	0.08	56	28
French Fries, fried potatoes	100	314.6	3.9	15.1	40.1	13	1.07	0.77	4	0.35	0	30
Potato (baked, boiled, or mashed)	100	100	2.1	0.1	23.3	5	0.41	0.33	15.1	0.32	0	12
Sweet potatoes or yams	100	108	1.3	0.3	25.4	17	0.95	0.16	10.8	0.06	458	8
Lettuce salad	100	14	0.9	0.1	3	18	0.41	0.15	2.8	0.04	25	29
Salad dressing	100	240	0.4	21.1	12.1	13	0.26	0.07	0.4	0.06	1.8	0
Mayonnaise	100	680	1	74.8	0.6	8	0.21	0.15	0	0.01	15.7	5

Food item	Amount (g)	Total Calories (kcal)	Protein (gm)	Total Fat (gm)	Carbs (gm)	Calcium (mg)	Iron (mg)	Zinc (mg)	Vitamin C (mg)	Vitamin B6 (mg)	Vitamin A (mcg)	Folate (mcg)
Chips (potato, corn, others)	100	547.5	7.2	36.4	53.5	0	1.28	0.95	21.5	0.2	1.7	84
Popcorn or pretzels	100	550.8	7.7	33	55.7	20.8	1.91	2.2	0.1	0.29	18.3	15
Crackers	100	421	9.5	8.9	74.3	21	5.13	0.82	0	0.06	0	221.9
Nuts	100	607	20	54	21	117	2.61	3.36	0.5	0.35	0.2	83
Cookies or brownies	100	474	5.1	23.3	63.9	26	3.2	0.65	0	0.03	0	69.3
Cake or cupcake	100	379	3.2	17.8	55.4	32	2.03	0.42	0	0	34.3	31.8
Pie	100	258.1	2.4	12.5	34.5	32.9	0.98	0.24	1.8	0.04	1.4	65.7
Jello	100	62	1.2	0	14.2	3	0.02	0.01	0	0	0	1
Chocolate or candy bar	100	535	7.7	29.7	59.4	189	2.35	2.3	0	0.04	59	11
Other candy (not chocolate)	100	394	0	0.2	62.9	3	0.3	0.01	0	0	0	0
Coffee or tea	100	1	0.1	0	0	2	0.01	0.02	0	0	0	2
Soda, soft drink, pop (not sugar free)	100	37	0.1	0	9.6	2	0.11	0.02	0	0	0	0
Soda, soft drink, pop (sugar free)	100	0.1	0	0	0	4	0.04	0	0	0	0	0
Beans(baked, chili, or other)	100	106	5.2	1.5	20	53	1.7	1.46	2	0.06	0.1	36
Rice	100	130	2.7	0.3	28.2	10	1.2	0.49	0	0.09	0	96.5
Spaghetti or other pasta	100	158	5.8	0.9	30.9	7	1.28	0.51	0	0.05	0	119.2
Pizza	100	266.6	12.5	11.3	29	262.5	1.65	1.35	2.4	0.12	65.5	96.9
Tacos, burritos	100	216	9.1	11.2	20	106	1.43	1.56	0.2	0.07	18.8	56.8
Macaroni and cheese	100	192.3	5.8	9	22.4	96.7	0.7	0.67	0	0.05	69.5	81.8
Hot dog	100	313	11.2	27.8	3.7	13	1.25	2.06	0	0.19	0	10
Sausage	100	339	19.4	28.4	0	13	1.36	2.08	0.7	0.33	12	3
Hamburger (prepared any way)	100	271	25.8	17.8	0	24	2.48	6.25	0	0.37	0	10
Canned Tuna	100	187	16	9.3	9.4	17	1	0.56	2.2	0.08	24.4	8
Fried fish, fish sticks	100	249	11	13.2	21.2	26	1.01	0.48	0	0.05	25	49.8
Other fish	100	105	22.8	0.9	0	14	0.49	0.58	1	0.28	14	8
Cold Cuts (baloney, ham, salami)	100	308	15.2	24.6	5.5	85	1.21	2.3	0.8	0.3	25	6
Fried Chicken, chicken nuggets	100	269	28.6	14.9	3.2	17	1.38	2.04	0	0.41	27	11.1
Other chicken or turkey	100	190	28.9	7.4	0	15	1.21	2.1	0	0.47	16	6
Pork or ham	100	222	24.4	13	0	28	0.68	2.15	0	0.64	2	0
Roast beef or steak	100	264	26.4	16.8	0	19	1.69	4.75	0	0.55	0	8
Liver, organ meats	100	175	26.5	4.7	5.2	6	6.17	5.23	0.7	1.03	7744	260
Peanut butter	100	588	25.1	50.4	19.6	43	1.87	2.91	0	0.54	0	74
Bread (slice) toast, roll, or pita	100	265	9.1	3.2	49.1	260	3.59	0.84	0	0.09	0	171.2
Butter (not margarine)	100	717	0.9	81.1	0.1	24	0.02	0.09	0	0	684	3
Margarine	100	0	0	0	0	0	0	0	0	0	0	0
Vegetable Soup	100	31	2.2	0.8	4.1	8	0.45	0.62	1	0.03	38.8	4
Other soup	100	25	1.3	0.9	3	6	0.66	0.16	0	0.02	15.2	12.2
Cornbread or tortillas	100	290.3	6	10.2	45.6	114.8	1.71	0.56	0.1	0.07	41.1	114.2
Eggs	100	143	12.6	9.5	0.7	56	1.75	1.29	0	0.17	160	47
Hot cereal or grits	100	71	2.5	1.5	12	9	0.9	1	0	0	0	6
Cold breakfast Cereal	100	379	13.1	6.5	67.7	52	4.25	3.64	0	0.1	0	32
Donut, fry bread	100	441.5	4.2	27	48	78.4	1.55	0.35	0	0.03	7.8	80.9

Food item	Amount (g)	Total Calories (kcal)	Protein (gm)	Total Fat (gm)	Carbs (gm)	Calcium (mg)	Iron (mg)	Zinc (mg)	Vitamin C (mg)	Vitamin B6 (mg)	Vitamin A (mcg)	Folate (mcg)
Sweet roll or muffin	100	371.1	4.9	16	52.8	46	1.36	0.52	0	0.07	5.5	130.4
Pancake, waffle, or French toast	100	227	6.4	9.7	28.3	219	1.8	0.56	0.3	0.18	53.2	56.2
English muffin or bagel	100	227	8.9	1.7	44.2	163	4	1.05	1.8	0.05	0	133.5
Biscuit	100	316.2	5.7	13.6	43.3	25.4	2.36	0.4	0.1	0.03	0.8	142.5

Note: Vitamin B6 = Pyridoxine; Vitamin A = Retinol Activity Equivalents; Folate = Dietary Folate Equivalents.
Source: Harvard T.H. Chan School of Public Health Nutrition Department's file download site. Available at: https://regepi.bwh.harvard.edu/health/nutrition.html (accessed on 11 December 2020).
