# Supplementary material for: Parenting Styles, Food Parenting Practices and Dietary Intakes of Preschoolers
Source: Nutrients. 2021 Oct 16;13(10):3630. doi: 10.3390/nu13103630 (PMC8537258; doi:10.3390/nu13103630)
Supplement: Supplementary file 1 [file nutrients-13-03630-s001.zip › nutrients-1385684-supplementary materials-2.pdf]

Table S1. The dimensions and items of the PDI-S survey used in this study.

Dimensions	Section	Items	Scoring Key
Nurturance	I	*The following statements represent matters of interest and concern to some parents. Not all parents feel the same way about them. Circle the number which most closely applies to you and your child.*<br><br>1) I encourage my child to talk about his or her troubles.[1]<br>4) My child and I have warm intimate moments together.[1]<br>7) I encourage my child to be curious, to explore, and to question things.[1]<br>9) I find it interesting and educational to be with my child for long periods.[1]<br>10) I make sure my child knows that I appreciate what he or she tries to accomplish.[1]<br>12) I respect my child's opinion and encourage him/her to express it.[1]	The dimension scores were computed by averaging responses to the items making up the dimension.
Amount of Control	III	*Listed below are pairs of statements concerning parents' attitudes toward childrearing. For each pair, read both statements. Then determine which statement you agree with most, and circle the letter in front of that statement. Circle ONLY ONE letter per item.*<br><br>1) A. Nowadays parents place too much emphasis on obedience in their children.<br>B. Nowadays parents are too concerned about letting children do what they want.<br>2) A. Children need more freedom to make up their own minds about things than they seem to get today.<br>B. Children need more guidance from their parents than they seem to get today.<br>3) A. I care more than most parents I know about having my child obey me.<br>B. I care less than most parents I know about having my child obey me.<br>4) A. I try to prevent my child from making mistakes by setting rules for their own good.<br>B. I try to provide freedom for my child to make mistakes and learn from them.<br>5) A. If children are given too many rules, they will grow up to be unhappy.<br>B. It is important to set and enforce rules for children to grow up to be happy adults.	Assign the parent a score of "1" for each time he or she chose the answers listed below. Count the total number of answers that match those listed. Score will range from 0 to 5 with higher score representing greater parental control.<br><br>1B, 2B, 3A, 4A, 5B

Note: 1. Response options: 1 = not at all like me, 2 = not much like me, 3 = somewhat like me, 4 = pretty much like me, 5 = very much like me, and 6 = exactly like me.

Source: Power TG. Parenting dimensions inventory-short version (PDI-S): a research manual. Unpublished manuscript. Washington State University; 2002.

Table S2. Food parenting practices exploratory factor analysis for Structure.

Scale Names and Items	Factor Loading	Internal Consistency
Meal and Snack Routines		0.74
• How many days of the week does your family sit at a table and eat dinner together?[a]	0.80	
• Does your family eat dinner while watching television?[b]	0.77	
• How often does your child eat in from of the TV?[c] (R)[d]	0.63	
• How often does your family eat a least one meal together each day?[e]	0.62	
• How often did you eat dinner with your child?[f]	0.57	
• How often did you have regularly scheduled meals and snacks with your family?[f]	0.55	
Modeling		0.73
• How often did your child see you eat when you were bored?[g] (R)[d]	0.86	
• How often did your child see you eat when you were angry or in a bad or sad mood?[g] (R)[d]	0.80	
• How often did you eat unhealthy snacks around your children?[g] (R)[d]	0.59	
• How often did your child see you eat late in the evening or at night?[g] (R)[d]	0.52	
• How often did you avoid buying sweets and chips or salty snacks and bringing them into the house?[f]	0.40	
• How often did your child see you eat while watching television, reading, or working?[g] (R)[d]	0.40	
• How often did you eat healthy meals or snacks while your child was around? ("healthy" defined as fruits, vegetables, lean meats, whole grains etc.)[f]	0.30	
Rules and Limits		0.70
Do you have the following food rules in your home...		
• How many snacks is your child allowed to eat?[h] (R)[d]	0.78	
• When to snack?[h] (R)[d]	0.77	
• Which snacks to eat?[h] (R)[d]	0.74	
• How many servings of fruit and vegetables your child should eat?[h] (R)[d]	0.51	
• No sweet snacks?[h] (R)[d]	0.16	
• No dessert except fruit?[h] (R)[d]	0.28	
Healthy Food Availability and Accessibility		0.46
• How often did you have the fruits, vegetables, and juices in your house?[f]	0.71	
• Based on the past 30 days, thinking about where you like to store food, please circle the appropriate response…fruits and vegetables in a place where they could be seen and easily reached.[f]	0.53	

Note: For Structure, higher responding scores reflect greater use of positive structure practices. a. Response options: one day or less = 0, 2 days = 0.17, 3 days = 0.34, 4 days = 0.51, 5 days = 0.68, 6 days = 0.85, and 7 days = 1. b. Response options: yes = 0 and no = 1. c. Response options: never = 1, 1 time or less per week = 0.75, 2-3 times per week = 0.5, 4-5 times per week = 0.25, and everyday = 0. d. (R) = items that reversed the coding. e. Response options: almost never = 0, sometimes = 0.33, often = 0.67, and almost always = 1. f. Response options: never = 0, rarely = 0.25, sometimes = 0.5, frequently = 0.75, and always = 1. g. Response options: never = 1, rarely = 0.75, sometimes = 0.5, frequently = 0.25, and always = 0. h. Response options: yes = 1 and no = 0.

Table S3. Food parenting practices exploratory factor analysis for Coercive Control.

**Scale Names and Items**	**Factor Loading**	**Internal Consistency**
Weight Concerns		0.79
• How concerned are you about your child having to diet to maintain a desirable weight?[a]	0.91	
• How concerned are you about your child becoming overweight?[a]	0.88	
• How concerned are you about your child eating too much when you are not around him/her?[a]	0.78	
• How much do you agree/disagree? I have to be sure that my child does not eat too much.[b]	0.59	
Restriction		0.75
How much do you agree/disagree?		
• I intentionally keep some foods out of my child's reach.[b]	0.80	
• If I did not guide or regulate my child's eating, he/she would eat too many junk foods.[b]	0.77	
• I have to be sure that my child does not eat too many sweets.[b]	0.77	
• I have to be sure that my child does not eat too much of his/her favorite foods.[b]	0.57	
Pressure to Eat		0.75
How much do you agree/disagree?		
• I have to be especially careful to make sure my child eats enough.[b]	0.86	
• If I did not guide or regulate my child's eating, he/she would eat much less than he/she should.[b]	0.80	
• If my child says "I am not hungry" I try to get him/her to eat anyway.[b]	0.70	
• My child should always eat all of the food on his/her plate.[b]	0.61	
Threats and Bribes		0.28
• How often did you use food as a reward for your child?[c]	0.81	
• How often did you use food as a punishment for your child?[c]	0.56	

Note: For Coercive Control which are negative behaviors, higher responding scores reflect greater use of those practices. a. Response options: unconcerned = 0, a little concerned = 0.25, concerned = 0.5, fairly concerned = 0.75, and very concerned = 1. b. Response options: disagree = 0, slightly disagree = 0.25, neutral = 0.5, slightly agree = 0.75, and agree = 1. c. Response options: never = 0, rarely = 0.25, sometimes = 0.5, frequently = 0.75, and always = 1.

Table S4. Food parenting practices exploratory factor analysis for Autonomy Support.

**Scale Names and Items**	**Factor Loading**	**Internal Consistency**
Child Involvement: Planning Meals		0.60
• How often did you plan meals/menus with your child?[a]	0.79	
• How often did you prepare meals with your child?[a]	0.78	
Child Involvement: Shopping		0.30
• How often do you take your child with your grocery shopping?[a]	0.71	
• Do you buy fruits and vegetables upon your child's request?[b]	0.85	
• Do you buy snacks or sugary cereal upon your child's request?[c] (R)[d]	0.05	

Note: For Autonomy Support, higher responding scores reflect greater child control for positive practices while lower scores reflect greater parent control. a. Response options: never = 0, rarely = 0.25, sometimes = 0.5, frequently = 0.75, and always = 1. b. Response options: not at all = 0, rarely = 0.25, sometimes = 0.5, quite a bit = 0.75, and very much = 1. c. Response options: not at all = 1, rarely = 0.75, sometimes = 0.5, quite a bit = 0.25, and very much = 0. d. (R)=items that reversed the coding.

Table S5. Multiple regression models for children's daily food group intake (without Autonomy Support variable).

Variables	Children' Daily Food Group Intake (servings/ 1000 kcal)											
	Fruits		Vegetables		Sweets		Dairy		Grain		Protein	
	Coef.[1]	P>|t|	Coef.	P>|t|	Coef.	P>|t|	Coef.	P>|t|	Coef.	P>|t|	Coef.	P>|t|
Model [2,3]	P = 0.0467* R^2 = 0.1298		P = 0.0005* R^2 = 0.1321		P = 0.0002* R^2 = 0.1517		P = 0.0218* R^2 = 0.1034		P = 0.0018* R^2 = 0.1023		P = 0.0136* R^2 = 0.1207	
Parenting Practices:												
Structure	0.999	0.032*	1.511	0.001*	-0.651	0.023*	-1.250	0.005*	0.610	0.349	0.609	0.028*
Coercive Control	0.316	0.508	0.714	0.114	0.367	0.161	-1.361	0.004*	0.119	0.738	0.310	0.219
Parenting Styles [4]:												
Authoritarian	-0.500	0.001*	-0.202	0.159	0.128	0.146	0.003	0.983	0.077	0.510	0.094	0.204
Indulgent	-0.595	0.001*	0.202	0.241	-0.145	0.152	0.115	0.538	0.079	0.507	0.184	0.094
Uninvolved	-0.358	0.101	0.199	0.299	-0.055	0.617	-0.070	0.757	-0.017	0.911	0.300	0.018*

Note: * $p < 0.05$. 1. Coef. = observed coefficients. 2. Results from separate bootstrapping regression models for each independent variable, adjusted for parent education level, child gender, and child BMI percentile category. 3. Adjusted regression models for children's daily intakes of fats (P = 0.0598, R^2 = 0.0812) were not significant. This result was not presented in the table. 4. The authoritative style served as the referent parenting style, meaning that the authoritarian, indulgent, and uninvolved feeding styles were compared against the authoritative feeding style in the model.

Table S6. Multiple regression models for children's daily food group intake (not adjusted on child's BMI percentile category).

Variables	Children' Daily Food Group Intake (servings/ 1000 kcal)									
	**Fruits**		**Vegetables**		**Sweets**		**Dairy**		**Grain**	
	Coef.[1]	P>|z|	Coef.	P>|z|	Coef.	P>|z|	Coef.	P>|z|	Coef.	P>|z|
Model [2,3]	P = 0.0271* R^2 = 0.1470		P = 0.0029* R^2 = 0.1151		P = 0.0185* R^2 = 0.0951		P = 0.0191* R^2 = 0.0970		P = 0.0002* R^2 = 0.1187	
Parenting Practices:										
Structure	0.849	0.088	1.249	0.020*	-0.656	0.033*	-1.292	0.008*	0.572	0.107
Coercive Control	0.364	0.448	0.503	0.252	0.543	0.034*	-1.364	0.010 *	0.013	0.962
Autonomy Support	0.955	0.178	0.896	0.107	0.240	0.498	0.096	0.890	-0.963	0.043*
Parenting Styles [4]:										
Authoritarian	-0.425	0.006 *	-0.158	0.293	0.160	0.087	0.026	0.866	-0.009	0.943
Indulgent	-0.568	0.001*	0.159	0.421	-0.082	0.435	0.162	0.420	0.023	0.854
Uninvolved	-0.293	0.171	0.225	0.230	-0.010	0.934	-0.042	0.846	-0.094	0.475

Note: * $p < 0.05$. 1. Coef. = observed coefficients. 2. Results from separate bootstrapping regression models for each independent variable, adjusted for parent education level and child gender. 3. Adjusted regression models for children's daily intakes of fats (P = 0.1770, R^2 = 0.0751) and protein (P = 0.1070, R^2 = 0.0950) were not significant. These results were not presented in the table. 4. The authoritative style served as the referent parenting style, meaning that the authoritarian, indulgent, and uninvolved feeding styles were compared against the authoritative feeding style in the model.
